# Update rules and semantic universals

**DOI:** 10.1007/s10988-022-09362-1

**Published:** 2022-08-29

**Authors:** Luca Incurvati, Giorgio Sbardolini

**Affiliations:** ILLC and Philosophy, University of Amsterdam, Amsterdam, The Netherlands

**Keywords:** Semantic universals, Monotonicity, Update semantics, Context change potentials, Lexicalization, Connectives, Quantifiers

## Abstract

We discuss a well-known puzzle about the lexicalization of logical operators in natural language, in particular connectives and quantifiers. Of the many logically possible operators, only few appear in the lexicon of natural languages: the connectives in English, for example, are conjunction *and*, disjunction *or*, and negated disjunction *nor*; the lexical quantifiers are *all, some* and *no*. The logically possible nand (negated conjunction) and Nall (negated universal) are not expressed by lexical entries in English, nor in any natural language. Moreover, the lexicalized operators are all upward or downward monotone, an observation known as the Monotonicity Universal. We propose a logical explanation of lexical gaps and of the Monotonicity Universal, based on the dynamic behaviour of connectives and quantifiers. We define update potentials for logical operators as procedures to modify the context, under the assumption that an update by $\phi$ depends on the logical form of $\phi$ and on the speech act performed: assertion or rejection. We conjecture that the adequacy of update potentials determines the limits of lexicalizability for logical operators in natural language. Finally, we show that on this framework the Monotonicity Universal follows from the logical properties of the updates that correspond to each operator.

## Background and motivation

Quantifiers in natural language are typically expressed by determiners such as *some, most, fewer than six*. A well-investigated property of lexical determiners is monotonicity. Let $\phi ,\psi$ be sets of individuals, with $\phi \subseteq \psi$.^1^

$$  \eqalign{ \hbox{ A } \hbox{ quantifier } \textsf{Q} \hbox{ is } \hbox{ upward } \hbox{ monotone } \hbox{ iff } \textsf {Q}_x\phi\,&\vDash\, \textsf {Q}_x\psi \cr \hbox{ downward } \hbox{ monotone } \hbox{ iff } \textsf {Q}_x\psi\,&\vDash\, \textsf {Q}_x\phi }<!endaligned>  $$

If *some* expresses the upward monotone quantifier $\exists$, then (1a) should entail (1b) since $\llbracket \text { run fast } \rrbracket \subseteq \llbracket \text { run } \rrbracket$, as is indeed the case. Similarly, if *no* expresses the downward monotone quantifier $\textsf {No}$ (the contradictory of $\exists$), we expect (2b) to entail (2a), as again is the case.

**Table:** 

1.	(a) Some students run fast (b) Some students run
2.	(a) No students run fast (b) No students run

A semantic universal is a generalization that explains some semantic properties of natural language expressions found across world languages (von Fintel and Matthewson, 2008). Barwise and Cooper’s (1981) monotonicity universal is the hypothesis, so far unfalsified, that lexical determiners express monotone quantifiers (Higginbotham and May, 1981; Keenan and Faltz, 1986; Keenan and Stavi, 1986; van Benthem, 1984). Moreover, monotone quantifiers are easier to learn, and this might explain why the monotonicity universal holds (Steinert-Threlkeld and Szymanik, 2019, 2020).

The monotonicity universal entails that a quantifier expressed compositionally in English by the phrase *exactly five* cannot be the denotation of a lexical entry in natural language, since (3a) does not entail and is not entailed by (3b).

**Table:** 

3.	(a) Exactly five students run fast (b) Exactly five students run

There are many more quantifiers in classical logic than are expressed in the lexicon of natural languages. But why so few? Many quantifiers definable in classical logic are not monotone, such as a hypothetical *exactly-five, and the monotonicity universal rules them out. But some monotone quantifiers are also missing. The canonical formulation of this observation is due to Horn (1972). The functions $\forall ,\exists$, No and Nall (the contradictory of $\forall$) form an Aristotelian Square of Oppositions along the dimensions of assertion/rejection and universal/particular. The corners are traditionally labelled by **A**, **E**, **I**, **O**. Across languages, only three corners of the square are ever occupied. For example, in English the lexicalized Aristotelian determiners are *every*, *some*, and *no*. A similar pattern applies to the connectives (*and, or, nor*), coordinated quantifiers (*both, either, neither*), and temporal adverbs (*always, sometimes, never*). Other languages pattern similarly (Carcassi and Sbardolini, 2022; Uegaki, 2022). 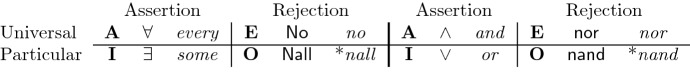


Consider the quantifier Nall in the **O** corner, the denotation of a hypothetical *nall. Since Nall is the contradictory of $\forall$, $\textsf {Nall}_x\phi$ is true iff $\forall _x\phi$ is false. Hence if $\phi \subseteq \psi$, then $\textsf {Nall}_x\psi\, \vDash\, \textsf {Nall}_x\phi$, and thus Nall is downward monotone. Therefore the monotonicity universal does not rule out *nall. However Nall must be expressed compositionally (*not all*), in English as in any other natural language.^2^

What might explain lexical gaps among logical operators? A quick look at the semantics does not indicate a relevant asymmetry between **A, I, E** operators and their **O** siblings. Horn (1989) and others have developed a Gricean account of lexical gaps, based on scalar implicatures. We will review the Gricean account in the next section, finding it unconvincing. Then we outline an alternative account based on the dynamics of information processing in context. According to our alternative account, gaps are explained not by scalar reasoning but by logic. It will not be the logic of classical truth-conditions, however, but a non-classical logic of dynamic update.^3^

A final preliminary remark. We understand lexical simplicity as monomorphemicity, following Keenan and Stavi (1986), and others. We flag here that the status of **E** expressions remains a matter of debate (Jespersen, 1917, p. 108). Split scope readings in Germanic languages appear to suggest that **E** expressions are not syntactically simple (Sauerland, 2000; Landman, 2004; Zeijlstra, 2011), though this point is controversial (De Swart, 2000; Geurts, 1996). In addition, in many languages and for many lexical categories, **E** operators follow the fate of **O** operators and are not lexicalized. This may indicate that further constraints than we discuss here might be at work. However, following Keenan and Stavi (1986), Horn (1989), Katzir and Singh (2013), and many others, we proceed under the reasonable assumption that there is a level of description of the linguistic phenomena at which a lexical distinction can be drawn between *nor*, *no*, *neither*, *never*, on the one hand, and *not and, not all, not both, not always* on the other.^4^

## The Gricean account

This section is a critical discussion of a fairly popular account of lexicalization gaps for logical operators, pioneered by Horn (1972, 1989) and recently extended by Katzir and Singh (2013) and Uegaki (2022). For brevity we focus on the four connectives of the Aristotelian Square.

An inventory of connectives *X* (a set of any of the connectives on the square) is said to ‘cover’ the same semantic space as another inventory *Y* just in case every truth-table expressed by *X* can be expressed by *Y* and *vice versa*. The Gricean account rests on two general principles. Let *X* and *Y* cover the same semantic space.

- The Gricean Condition: If $Y\subset X$, *X* cannot be lexicalized.
- The Negation Condition: If *X* contains more instances of negation than *Y*, *X* cannot be lexicalized.

The Gricean Condition rules out the full set $\{$and, or, nor, *nand $\}$, because there is a scalar implicature from **I** to **O**. For example, (4a) implicates (4b).

**Table:** 

4.	(a) She is either at school or at work (b) She is not both at school and at work

Thus the **O** corner, which would be expressed by a hypothetical *nand, is already covered by *or*. Since the inventory $\{$*and, or, nor* $\}$ is smaller, the full set cannot be lexicalized. Presumably however, there is also a scalar implicature in the opposite direction, from **O** to **I**. Thus the Gricean Condition allows for two candidate inventories, $\{$*and, or, nor* $\}$ and $\{$and, *nand, nor $\}$. This is where the Negation Condition comes in. Since the latter inventory contains more negations than the former, it cannot be lexicalized, and the Gricean account predicts the attested pattern. The Gricean account is intended to apply to the quantifiers too and can be further refined (Katzir and Singh, 2013), and extended beyond the Aristotelian Square (Uegaki, 2022). There are important differences between different versions of the Gricean account but our doubts arise at this early stage. We have two main concerns.

First, the notion of ‘covering’ is employed arbitrarily. The reasoning above implicitly assumes that the only means of covering a semantic space are lexicalization and scalar implicature. In natural language, a semantic space may be covered by implicature, by dedicated lexical items, but also compositionally. The material conditional, for example, is ordinarily expressed in English by disjunction and negation: *either not*-*p* *or* *q*. Therefore, compositional combination of lexical operators must be a valid means of covering a semantic space. If so, a language whose only connective is *nand covers the same semantic space as $\{$*and, or, nor* $\}$ because the former inventory can express all Boolean truth-tables by compositional means (that is, $\{*{nand}\}$ is truth-functionally complete). Moreover, it would seem that $\{$*nand $\}$ contains as many negations as $\{$*and, or, nor* $\}$, both including only one “negative”. If there is a preference for economy by which languages tend to lexicalize less (which arguably justifies the Gricean Condition), then $\{$*nand $\}$ should be preferred to the attested inventory $\{$*and, or, nor* $\}$ by reasons of economy. The Gricean account runs into difficulties if we drop an arbitrary restriction to scalar implicature as the only means of expressing a meaning besides lexicalization.

Secondly, we have doubts about the Negation Condition. On what grounds should we say that $\{$and, *nand, nor $\}$ contains more negations than $\{$*and, or, nor* $\}$? The idea seems to be that *nand contains an instance of negation that *or* does not contain, for the two inventories are otherwise equivalent. But does it? The question turns on the primitive resources of the metalanguage used to encode the truth-conditions of natural language expressions. Consider a metalanguage in which nand and nor are the only primitives. With respect to such hypothetical metalanguage, *nand and *nor* would not contain any negations except perhaps themselves: they express the semantic primitives of the chosen metalanguage after all. On the contrary, it’s *or* that would contain a negation, since it would be expressed as negated nor in such a metalanguage. Thus, it is only with respect to a specific choice of metalinguistic primitives that $\{$and, *nand, nor $\}$ contains more negations than $\{$*and, or, nor* $\}$. With respect to a different semantic metalanguage, the Negation Condition would not make the right predictions.

In a recent version of the Gricean account, Katzir and Singh (2013) assume that the primitives of the semantic metalanguage are the basic operations of a classical Boolean algebra: *inf* $\sqcap$, *sup* $\sqcup$, and negation. Similarly, the metalanguage of Uegaki (2022) is the standard language of propositional logic ($\lnot , \wedge , \vee$). With respect to such primitives, there is no doubt that $\{$and, *nand, nor $\}$ contains more negations than $\{$*and, or, nor* $\}$. But the conclusion assumes what was supposed to be explained, namely that the primitives are conjunction and disjunction (or *inf* and *sup*), as opposed to nand.

We have two reasons to be skeptical of the Gricean account: the notion of ‘covering’ is curtailed arbitrarily and the use of metalinguistic primitives hides a circularity. We set out to look for another account. An appropriate choice of primitives will be crucial to our explanation below, as our second criticism of the Gricean account emphasizes. In Sect. 3, we will motivate the choice of primitives as basic operations of context update in a dynamic setting in which sentences can modify the context by being asserted or rejected. We will then discuss connectives and quantifiers in Sects. 4 and 5 respectively. As we will finally show in Sect. 6, our account has the additional advantage over the pragmatic account that it predicts Barwise and Cooper’s (1981) monotonicity universal. In contrast, the Gricean account simply assumes that the operators belonging to the Aristotelian Square are monotone. Our account predicts that the lexicalized operators are upward or downward monotone, depending on whether their arguments are asserted or rejected. Thus we think that considerations of explanatory generality weigh in favour of our proposal.

## Update potentials and force

The lexicalization patterns of logical operators appear puzzling in part because classical logic, which we ordinarily use to encode semantic values, greatly outstrips the expressive power of the lexicon of natural language. Classical logic makes many more distinctions than the lexicon does, and thus the problem arises to explain away what’s missing. Horn’s strategy is to keep the logic classical and to invoke Gricean pragmatics to bridge the gap. Our strategy is to revise the logic and make the puzzle disappear: the primitives of the logical language we define below express what the lexicon of natural language can express, while semantic values that are not lexicalized can be recovered by compositional combination of lexicalized material, just like in natural language.

We begin with a formal language $L_{\rm {Bool}}$ closed under the signature below, and for now we continue focusing on the Aristotelian Square for simplicity. In Sect. 4.3 we will extend $L_{\rm {Bool}}$ by adding negation, and in Sect. 5 we will add the quantifiers.

$$  \eqalign{ \phi :=\ p\ |\ \phi \wedge \phi \ |\ \phi \vee \phi \ |\ \phi \,\textsf{ nor }\,\phi \ |\ \phi \,\textsf { nand }\,\phi }<!endaligned>  $$

From a static perspective, a declarative sentence (relative to a context) represents the world as being a certain way. In an intensional framework the static semantic value of an atomic sentence *p* is the set of worlds $\llbracket p \rrbracket ^{c,g}\subseteq W$ in which the sentence is true. The semantic values of complex sentences are calculated compositionally in the familiar way. (Superscripts on interpretation are omitted for the rest of this section, for simplicity.)

### **Definition 1**

Static Interpretation

**Table:** 

$\llbracket \phi \wedge \psi \rrbracket =\llbracket \phi \rrbracket \cap \llbracket \psi \rrbracket$	$\qquad \llbracket \phi\,\, \textsf { nor }\,\,\psi \rrbracket =(W\backslash \llbracket \phi \rrbracket )\cap (W\backslash \llbracket \psi \rrbracket )$
$\llbracket \phi \vee \psi \rrbracket =\llbracket \phi \rrbracket \cup \llbracket \psi \rrbracket$	$\qquad \llbracket \phi \,\,\textsf { nand }\,\,\psi \rrbracket =(W\backslash \llbracket \phi \rrbracket )\cup (W\backslash \llbracket \psi \rrbracket )$

The static semantics gives no indication why, of the operators in $L_{\rm {Bool}}$, nand is not lexicalized while the others can be. After all, the semantic metalanguage does have the expressive resources to encode the interpretation of nand, if combinations of intersection, union, and complement can be expressed lexically. Perhaps considerations other than truth-conditions could help us move closer to a principled solution to this puzzle.

From a dynamic perspective, declarative sentences update the context in which they are uttered by modifying the information it contains. In dynamic semantics, an atomic sentence *p* modifies an initial context *c* into an updated context *c*[*p*]. Update potentials are then extended to complex sentences compositionally. However, in Heim’s (1983, 1990) semantics, just as in static semantics, update potentials are calculated by combining unions and complements. Thus the update potential of nand is straightforwardly definable as $(c\backslash c[\phi ])\cup (c\backslash c[\psi ])$. The same observation applies to Veltman’s (1996) semantics. 
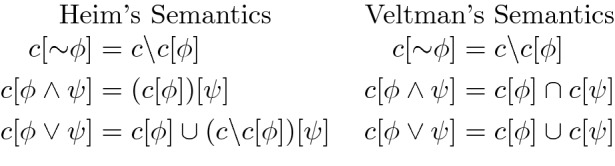
This is not surprising, since these theories were not designed to explain semantic universals. Notice, however, a difference between Heim and Veltman. Heim calculates the update potential of conjunction by directly combining the update potentials of the conjuncts: the input context *c* is first updated to an intermediate context $c[\phi ]$, which is then updated to a final context. For Veltman the update potential of conjunction is expressed as a relation between contexts (intersection). Heim’s update rule for conjunction operates recursively on a single input context, whereas for Veltman two separate updates are calculated and then combined by intersecting their outputs. We think that the number of times the context *c* is fed as an input to an update process is not merely a matter of stylistic presentation, but a matter of computational complexity. On our version of dynamic semantics, updates are context change operations that conform to Heim’s standard for conjunction as far as possible. Under natural assumptions, we will show that all connectives in $L_{\rm {Bool}}$ can be encoded as operations on a single input *c*, except for nand. An update encoding the latter requires the input twice: an additional complexity that is revealed by our formalism. We submit that this observation is key to explain *nand’s failure to lexicalize.^5^ In the second part of the paper, a natural extension of the account explains why *nall is not a lexical determiner.

### Overview of the account

The purpose of this section is to give a brief overview of our account before digging in the details. We make two assumptions that are not in Heim or Veltman. Firstly, our system is bilateral: assertion and rejection are both primitive speech acts, which can be modelled as intersection $\cap$ and complement $\backslash$ respectively. In Veltman and Heim, the relationship between context *c* and a proposition $\phi$ is always modelled by intersection. Complement comes in for negation, but as an operation between contexts. This reflects the assumption, implicit in Veltman's and Heim’s systems, that context update always proceeds by way of assertion. Secondly, we allow for updates not only by means of a single proposition, but of more propositions at the same time. In particular, by asserting a disjunction the context will be updated with information that belongs to either disjunct.

On the basis of these two assumptions, a set-theoretic interpretation of the assertion $$  +  $$ of atomic formulas, and of their conjunctions and disjunctions, can be given as follows. Let a context $$  C\subseteq W  $$ be a set of possible worlds. Let $$  p, p_1, p_2,  $$ be atomic formulas.

$$  \eqalign{ C[+p]&= C\cap \{w\in \llbracket p \rrbracket \}\cr C[+p_1\wedge p_2]&= (C\cap \{w\in \llbracket p_1 \rrbracket \})\cap \{w\in \llbracket p_2 \rrbracket \}\cr C[+p_1\vee p_2]&= C\cap \{w\in \llbracket p_i \rrbracket :i\in (1,2)\} }<!endaligned>  $$

Assertion of *p* is just intersection of its content with context, as for Stalnaker (1978). Assertion of conjunction is a sequence of assertions of the conjuncts, as for Heim (1983). Finally, assertion of disjunction is intersection with the set of worlds that verify at least one disjunct. Note that the case of disjunction is where we employ the assumption that context can be updated with the information of more than one sentence at once (as we discuss in the Appendix, this rule is equivalent to Veltman’s (1996) disjunction over the assertoric fragment of the language). In these definitions, a context is a set of worlds that interacts with classical propositions (sets of possible worlds) and updates are processes that operate on an input *C*, occurring only once, as in Heim’s standard on conjunction.

Rejection is the same as assertion, except for the relation between context and content, which can be modelled as set-theoretic subtraction.

$$  \eqalign{ C[-p]=C\backslash \{w\in \llbracket p \rrbracket \} }<!endaligned>  $$

Rejection is a negation-like operator, but it is not negation properly speaking: rejection is a force marker, and specifically a relation between context and the information carried by a sentence. Negation properly speaking is not just an operator on force, but on content, and will come in later. With rejection, however, we can formulate some negative updates by switching intersection to subtraction in the assertoric rules of conjunction and disjunction.

$$  \eqalign{ C[-p_1\wedge p_2]&= (C\backslash \{w\in \llbracket p_1 \rrbracket \})\backslash \{w\in \llbracket p_2 \rrbracket \}\cr C[-p_1\vee p_2]&= C\backslash \{w\in \llbracket p_i \rrbracket :i\in (1,2)\} }<!endaligned>  $$

Rejection of conjunction has the same structure as assertion of conjunction: consecutive updates, but with subtractions instead of intersections. Consequently, $C[-p_1\wedge p_2]=(C\backslash \llbracket p_1 \rrbracket )\cap (C\backslash \llbracket p_2 \rrbracket )$, which are the truth-conditions of nor. Rejection of disjunction has the same structure as assertion of disjunction: a single update with worlds from either disjunct, but with subtraction in place of intersection. As a result, $C[-p_1\vee p_2]=(C\backslash \llbracket p_1 \rrbracket )\cap (C\backslash \llbracket p_2 \rrbracket )$. Once again we obtain the truth-conditions of nor. Therefore *p* nor *q* may be expressed as rejected disjunction $-p\vee q$, with its expected truth-conditions. However *p* nand *q* may not be expressed as rejected conjunction, for the latter does not determine the truth-conditions of nand. Here we find that the classical duality of conjunction and disjunction is broken.

Of course, there is a way of expressing nand as an update. What is needed is negation $\sim$, as defined by Heim and Veltman as an operator on contexts:

$$  \eqalign{ C[+{\sim }\phi ]&= C\backslash C[+\phi ] }<!endaligned>  $$

We call this operator complement negation, and remark that it is not an operation on force: its arguments are not a context and a proposition (as for assertion and rejection) but two contexts. Indeed, the variable *C* occurs twice in its definition. We also follow Veltman and Heim in thinking that the dynamic contribution of the word *not* (or its analog in other languages) is characterized by complement negation $\sim$. With complement negation, we may calculate the contribution of negated conjunction straightforwardly, $C[+{\sim }(p\wedge q)]=C\backslash C[+p\wedge q]$, with its expected truth-conditions. Therefore *p* nand *q* can now be expressed in the update system as negated conjunction.

Our notation is designed to keep track of occurrences of the context variable. Its occurrences reveal implicit assumptions about the computational processes underlying an update. With complement negation the input context *C* is taken not only once but twice. First, to calculate an update, and then to subtract the result from the initial input. This is an extra layer of complexity that is made explicit by our formalism. We contend that this additional complexity is visible in natural language at lexical level. For natural language expresses the complement of conjunction not as a simple lexical entry but by combining *and* with *not*, just as in our system we have to combine $\wedge$ and $\sim$.

In addition, we contend that the expressive power of the lexicon for the binary connectives is characterized by the assumptions we have made concerning $\wedge$ and $\vee$: updates are procedures that operate on an input context and at least one (but possibly more) propositions. On the basis of the same assumptions we can define nor. By adding $\sim$ all Boolean meanings including nand can be expressed by compositional combination of the lexical material. However, adding $\sim$ means adding lexical negation *not*, and thus the possibility of calling the input more than once in computing the update of a complex sentence. Thus, on our account, the lexicon of natural language does not include nand because nand is too complex to be expressed by asserting and rejecting propositions on a single context as input. It can only be expressed compositionally by means of a specialized operation on contexts, which is what *not* provides. The update system we outline makes the extra complexity of nand explicit in the logical form of an update.

Our explanation of lexical gaps rests on two assumptions, which can be modelled set-theoretically in the way we have shown. But the modelling choices are not essential to the ideas we presented. The two assumptions we need may be motivated independently. The first assumption is bilateralism: assertion $+$ and rejection − are both primitives. In particular, the latter is not equivalent to the assertion of negation. Assertion and rejection have different effects on the dynamics of conversation. Rejection is typically signalled by response particles (*No!*), headshakes, or manifestations of dissent. Some languages have dedicated expressions for sentential rejection (Frana and Rawlins, 2019). There is a long tradition of bilateralism in logic (Smiley, 1996; Rumfitt, 2000; Incurvati and Schlöder, 2017; Berto and Restall, 2019). Reasons against conflating rejection with the assertion of negation are found in linguistics (Horn, 1989; De Swart, 2010), philosophy (Price, 1990), and cognition studies (Dimroth, 2010). We do not summarize them here.

The second assumption we introduce, which goes beyond standard update semantics, concerns the mechanics of update. We allow for multiple propositions to contribute information to a context, not only one by one, but jointly in a single update. This is indispensable for our account of disjunction. The status of disjunction is a matter of some debate in linguistics, both statically (Ciardelli et al., 2018; Simons, 2001; Groenendijk and Stokhof, 1984; Alonso-Ovalle, 2006) and dynamically (Soames, 1989; Schlenker, 2009; Rothschild, 2011). We think our thesis about lexicalization is compatible with the main competing views, and leave some remarks for the Appendix.^6^

### Update proposal and execution of update

The purpose of this section is to present a conception of context and of update that justifies the innovations we introduced to explain the missing nand. According to Stalnaker (1978), a conversation is a process of collective belief revision, during which interlocutors engage in the task of finding out which world is actual. Interlocutors provide information to update their context (the set of possibilities they all jointly presuppose). An update is a transition from the an initial state of the context to a next state.^7^ On this view, updates are brought about by speech acts such as assertion. According to Stalnaker, an assertion comprises two stages: first, there is a proposal made by the speaker to add information to the context; once the proposal is accepted, the update comes into effect with the elimination from the initial context of the possibilities that are incompatible with the proposal. The two stages are separate because, as Stalnaker (1978, p. 86) indicates, the proposal might be rejected. The need to distinguish the proposal and the execution of an update has been recognized in the literature on questions by Ginzburg (1996), Farkas and Bruce (2010), and many others.

Stalnaker’s conception of conversation has room for the assumptions we introduce. Bilateralism comes in at the first stage: a speaker proposes to accept or reject some possibilities. The first case characterizes the speech act of assertion, the second rejection. In addition, nothing in the Stalnakerian view prevents the possibilities highlighted in the proposal phase from coming from more than one sentence. Of course, only one sentence is operational if the utterance is atomic, but complex sentences may be uttered. In this case, one update may be performed with many sentential constituents contributing to it.

In order to model these assumptions, we now take contexts to be ordered sets of possibilities (as it is sometimes done in the belief revision literature, see van Benthem, 2007). The order indicates that belief is not an all-or-nothing affair: some possibilities may be more likely than others.

Let a context be a pair $c=(C,\le )$ of a set of worlds $C\subseteq W$, typically non-empty, and a total pre-order $\le$ on them (a reflexive and transitive relation, in which any two worlds are comparable and there are no cycles). The order reflects the interlocutors’ opinions about which worlds are likely to be actual: for any *w* and *v*, $w\le v$ if, and only if, *w* is at least as likely to be the actual world as *v*. The proposal re-arranges the worlds in the initial context according to their likelihood, and an update comes into effect when the initial possibilities are restricted to the more likely ones only. For example, if *p* is asserted in context *c*, the proposal is to place all the *p*-worlds at least as low in the ordering as the non-*p*-worlds: the latter are deemed less likely than the former. In the second step, the less likely worlds are eliminated from the context. Consequently, $c[+p]$ includes the *p*-worlds and not the non-*p*-worlds.

We calculate how speech acts change a context by combining two steps. The first step is an update proposal ${{prop}}$. This is a function that takes a context *c* with its order $\le$ and a proposition $\phi$ and defines a new context which is the same as *c* except for a new order in which the $\phi$-worlds are more or less likely than they initially were. (Proposals are ‘lexicographic updates’ in the sense of van Benthem, 2007.) The second step is an update execution ${{exec}}$. This function takes a context as input and defines a (typically, smaller) context in which the less likely worlds of the input are eliminated. We begin by introducing updated likelihoods on worlds.

#### **Definition 2**

**New Likelihoods**. Let $c=(C,\le )$ be a context and $\phi$ a sentence.

- For all *w* and *v* in *C*, let $w\le ^{+\phi }v$ iff $w\in \llbracket \phi \rrbracket$ and $v\not \in \llbracket \phi \rrbracket$, or else $w\le v$.
- For all *w* and *v* in *C*, let $w\le ^{-\phi }v$ iff $w\not \in \llbracket \phi \rrbracket$ and $v\in \llbracket \phi \rrbracket$, or else $w\le v$.

The ‘or else’ clause is short for the following: $w\le ^{+\phi }v$ iff $w\in \llbracket \phi \rrbracket$ and $v\not \in \llbracket \phi \rrbracket$, or else (if $w\in \llbracket \phi \rrbracket$ and $v\in \llbracket \phi \rrbracket$, or if $w\not \in \llbracket \phi \rrbracket$ and $v\not \in \llbracket \phi \rrbracket$, then) $w\le v$. In other words, $\le ^{+\phi }$ is the union of two relations, and for any two worlds *w* and *v*, $w\le ^{+\phi }v$ just in case either $w\le v$ by the input-given relation $\le$, or *w* verifies $\phi$ but *v* doesn’t. Similarly for $\le ^{-\phi }$, *mutatis mutandis*. We use these updated likelihoods to define a proposal function ${{prop}}$ from context to context.

#### **Definition 3**

**Positive and Negative Proposals**. Let $c=(C,\le )$ be a context and *p* an atomic sentence.

- $$  {{prop}}^{+p}(c):=(C,\le ^{+p})  $$
- $$  {{prop}}^{-p}(c):=(C,\le ^{-p})  $$

A positive proposal takes a context $c=(C,\le )$ and defines a new context $(C,\le ^{+p})$ with its ordering upgraded. Similarly for a negative proposal. With $\le ^{+p}$, the *p*-worlds become at least as likely as the non-*p*-worlds, while the rest remains unchanged. With $\le ^{-p}$ the *p*-worlds become less likely than the non-*p*-worlds, leaving the rest unchanged. In these definitions, proposals are restricted to the possibilities compatible with atomic sentences. Proposals for complex sentences are not defined but calculated (see below).

Next, we define the execution function *exec*.

#### **Definition 4**

**Execution**. Let $c=(C,\le )$ be a context. Let $\text {min}(c)=\{w\in C: \forall v\in C(w\le v)\}$.

- $$  {{exec}}(c):=(C\cap \text {min}(c),{\le }|^{C\cap \rm {min}(c)})  $$

The execution operator ${{exec}}$ takes a context and restricts it to its minimal worlds. The set of worlds *C* is restricted to the more likely worlds, and the order is restricted accordingly: ${\le }|^{C\cap \rm {min}(c)}$ indicates that the field of the relation $\le$ is restricted to $C\cap \text {min}(c)$.

On the basis of these definitions we can introduce assertion and rejection of atomic sentences. An update by assertion or rejection consists of at least one proposal and execution of it.

#### **Definition 5**

**Assertion and Rejection of atomic sentences**. Let $c=(C,\le )$ be a context.

- $$  c[+p]:={{exec}}({{prop}}^{+p}(c))  $$
- $$  c[-p]:={{exec}}({{prop}}^{-p}(c))  $$

Given a context $c=(C,\le )$, the execution ${{exec}}$ applies to a context, $(C,\le ^{+p})$ or $(C,\le ^{-p})$, that differs from the initial context *c* only by its worlds having been re-arranged to upgrade or downgrade the *p*-worlds. Assertion and rejection of atomic sentences are thus executed proposals.

Suppose for simplicity that the conversation begins with a “blank slate” context, all of whose worlds are equally likely to be actual. Suppose that *p* is asserted in such context. First, ${{prop}}^{+p}(C,\le )=(C,\le ^{+p})$ is defined: all the *p*-worlds become at least as likely as the non-*p*-worlds, and within those two zones the worlds remain equally likely. Then the execution ${{exec}}$ is applied to $(C,\le ^{+p})$ and the less likely worlds are eliminated: in this case, those incompatible with *p*. The ordering is reversed in case of rejection, with the worlds incompatible with *p* now placed among the minimal ones. As a result, $c[-p]$ contains only worlds in *c* that are incompatible with *p*.

An important benchmark for standard dynamic systems is the equivalence between the update generated in *c* by an assertion of $\phi$, and the intersection of the context with the static meaning of $\phi$. This equivalence is sometimes called Stalnaker’s Rule of Assertion. Since we are assuming that sentences are nowhere undefined, the Rule is valid in the bilateral system for atomic formulas, and takes the following form (to accommodate for the fact that contexts are ordered sets).

**Minimal adequacy:** Atomic sentences. $(C,\le )[+p] = (C\cap \llbracket p \rrbracket ,\le )$

In this sense, assertion corresponds to intersection. Stalnaker’s Rule is about what possibilities are “live” for the interlocutors. Because of how we have defined the new orderings in Definition 2, the order of the new context is always a restriction of the old one. In addition, rejection corresponds to complement: $(C,\le )[-p]=(C\backslash \llbracket p \rrbracket ,\le )$.

We will take Stalnaker’s Rule of Assertion as a condition of minimal adequacy for update potentials. It is ‘minimal’ because it is restricted to atoms throughout, since the *prop* function is defined only on atoms. Next, we will introduce update potentials for complex sentences, developing the idea that updates are procedures to change the context in which possibilities of one or more sentences are upgraded or downgraded, and then discarded.

## The connectives

In this section we discuss the familiar Boolean connectives of $L_{\rm {Bool}}$. The primitives we have introduced do not provide an adequate definition for the update potential of nand, but do account for $\wedge ,\vee ,\textsf{nor}$.

### Assertion of conjunction and disjunction

For the assertion of conjunction, we follow Heim’s (1983) insight. A local context is defined once the first proposal is made, followed by a second proposal and the definition of the final context.

#### **Definition 6**

**Assertion of Conjunction.** Let $c=(C,\le )$ be a context.

- $$  c[+p\wedge q] := {{exec}}({{prop}}^{+q}({{exec}}({{prop}}^{+p}(c))))  $$

**Fig. 1:**
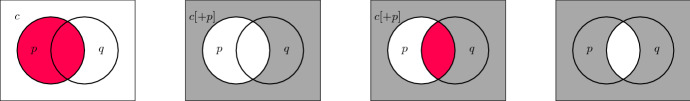
Assertion of conjunction. (1) The *p*-worlds in *c* are at least as likely as the non-*p*-worlds (red); (2) an intermediate context $c[+p]$ is defined (white) by eliminating the less likely worlds; (3) the *q*-worlds in $c[+p]$ are at least as likely as the non-*q*-worlds; (4) the updated context is defined (white)

Assertion of conjunction is simply a sequence of assertions, one for each conjunct. The procedure is illustrated in Fig. 1. Unpacking the definitions, assertion of conjunction takes four steps.

1. ${{prop}}^{+p}(c)$ is defined: the *p*-worlds in *c* become at least as likely as the non-*p*-worlds;
2. ${{exec}}({{prop}}^{+p}(c))=c[+p]$ is defined: the less likely non-*p*-worlds are eliminated;
3. ${{prop}}^{+q}({{exec}}({{prop}}^{+p}(c)))$ is defined: the *q*-worlds in $c[+p]$ become at least as likely as the non-*q*-worlds;
4. finally, ${{exec}}({{prop}}^{+q}({{exec}}({{prop}}^{+p}(c))))$ is defined: the non-*q*-worlds are eliminated.

Thus ‘$c[+p\wedge q]$’ can be also written ‘$(c[+p])[+q]$’. As a result, the final context contains only worlds that verify both *p* and *q*. In this procedure, a local context $c[+p]$ intervenes, and the second proposal by *q* is made about the local context. Consequently, assertion of $p\wedge q$ amounts to restricting the set of possibilities *C* of the initial context to $(C\cap \{w:w\in \llbracket p \rrbracket \})\cap \{w:w\in \llbracket q \rrbracket \}$, and minimal adequacy holds.

**Minimal adequacy:** Conjunction. $(C,\le )[+p\wedge q]=(C\cap \llbracket p\wedge q \rrbracket ,\le )$

The difference between conjunction and disjunction in the current setting is straightforward: disjunction defines no local context after a proposal is made for the first argument, and thus the information of both *p* and *q* modifies the same context without order effects.

#### **Definition 7**

**Assertion of Disjunction.** Let $c=(C,\le )$ be a context.

- $$  c[+p\vee q] := {{exec}}({{prop}}^{+q}({{prop}}^{+p}(c)))  $$

In this case, two proposals are made in the same initial context: the first pushes the *p*-worlds low in the ranking; the second takes place in the same context (no worlds are eliminated yet) and pushes the *q*-worlds low in the ranking. Thus the minimal worlds verify *p* or verify *q*. Worlds that are neither *p* nor *q* are eliminated when the update is finally executed. There are thus three steps:

1. ${{prop}}^{+p}(c)$ is defined: the *p*-worlds in *c* become at least as likely as the non-*p*-worlds;
2. ${{prop}}^{+q}({{prop}}^{+p}(c))$ is defined: the *q*-worlds become at least as likely as the non-*q*-worlds;
3. ${{exec}}({{prop}}^{+q}({{prop}}^{+p}(c)))$ is defined: the neither-*p*-nor-*q*-worlds are eliminated.

**Fig. 2:**
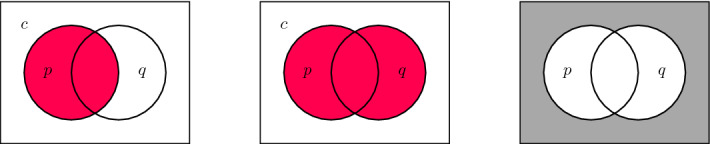
Assertion of disjunction. (1) The *p*-worlds in *c* are at least as likely as the non-*p*-worlds; (2) the *q*-worlds in *c* are at least as likely as the non-*q*-worlds; (3) the updated context is defined

In an update by disjunction, the information carried by the disjuncts is collected together, and then a single execution operation is performed. The relation of relative likelihood $\le ^{+\phi }$ is defined (Definition 2) as the union of two relations: one is the contextually given $\le$ and the other says that $\phi$-worlds are “better”. As a consequence, for any worlds *w* and *v* in ${{prop}}^{+q}({{prop}}^{+p}(c))$, *w* is at least as likely as *v* if $w\in \llbracket p \rrbracket$ and $v\not \in \llbracket p \rrbracket$, or if $w\in \llbracket q \rrbracket$ and $v\not \in \llbracket q \rrbracket$, or else if $w\le v$.^8^ The rule for disjunction is illustrated in Fig. 2 and has the consequence that the set *C* of initial possibilities of the context is restricted to $C\cap \{w\in \llbracket p_i \rrbracket :i\in (1,2)\}$. Therefore the rule is minimally adequate.

**Minimal adequacy:** Disjunction. $(C,\le )[+p\vee q] = (C\cap \llbracket p\vee q \rrbracket ,\le )$

In the updates we have described for conjunction and disjunction, as with Heim’s standard for conjunction, the initial context *c* is taken as input only once at the beginning. The difference between conjunction and disjunction is a matter of whether a local context is defined after a first proposal is made. In the case of disjunction, no local context is defined, but the procedure continues with another proposal which is appended to the proposal of the first sentence.

### Rejection of conjunction and disjunction

From the perspective of the present paper, assertion and rejection are on a par. As we have seen in the previous section, assertion and rejection of atomic formulas differ only in the polarity of the proposal ($+$ or −). It is natural therefore to define negative updates for conjunction and disjunction by switching the polarity of the proposal: possibilities that in a positive update “move down”, in a negative update “move up”, so to speak. It will appear that rejections of conjunction and disjunction, so defined, are truth-conditionally equivalent and adequate for nor but not for nand. We begin with rejection of disjunction.

#### **Definition 8**

**Rejection of Disjunction.** Let $c=(C,\le )$ be a context.

- $$  c[-p\vee q]:={{exec}}({{prop}}^{-q}({{prop}}^{-p}(c)))  $$

**Fig. 3:**
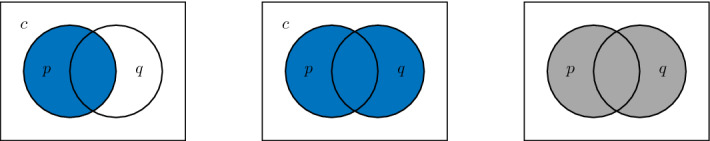
Rejection of disjunction. (1) The *p*-worlds in *c* are less likely than the non-*p*-worlds (blue); (2) the *q*-worlds in *c* are less likely than the non-*q*-worlds; (3) the updated context is defined (white)

The procedure is illustrated in Fig. 3.

1. ${{prop}}^{-p}(c)$ is defined: the *p*-worlds in *c* become less likely than the non-*p*-worlds;
2. ${{prop}}^{-q}({{prop}}^{-p}(c))$ is defined: the *q*-worlds in *c* are added to the less likely worlds;
3. ${{exec}}({{prop}}^{-q}({{prop}}^{-p}(c)))$ is defined: the proposals are jointly executed and the less likely worlds are eliminated.

A proposal is made according to which possibilities compatible with *p* are less likely than those incompatible with *p*. Without eliminating any worlds yet, a similar proposal is made about *q*. The minimal worlds are now those neither in *p* nor in *q*. Consequently, $-p_1\vee p_2$ restricts the set *C* of initial possibilities in context *c* to $C\backslash \{w\in \llbracket p_i \rrbracket :i\in (1,2)\}=(C\backslash \llbracket p_1 \rrbracket )\cap (C\backslash \llbracket p_2 \rrbracket )$.

The simplest hypothesis is that assertions of *p* *nor* *q* update the context as rejected disjunctions. Conversely, rejections of *p* *nor* *q* are tantamount to assertions of disjunction.

#### **Definition 9**

**Assertion and Rejection of Nor**. Let $c=(C,\le )$ be a context.

- $$  c[+p \, \textsf {nor} \, q]:= c[-p\vee q]  $$
- $$  c[-p \, \textsf{ nor } \, q] := c[+p\vee q]  $$

Given the truth-conditions of nor, it follows that with these rules nor is minimally adequate.

**Minimal adequacy:** Nor. $(C,\le )[+p\,\textsf { nor }\,q]=(C\cap \llbracket p\,\textsf { nor }\,q \rrbracket ,\le )$

We modelled the difference between conjunction and disjunction as a matter of there being an intermediate local context. Thus for $-p\wedge q$ a proposal is made according to which possibilities compatible with *p* are less likely than the others. A local context is generated, which includes the minimal worlds, hence those incompatible with *p*. With respect to this context, a proposal is made according to which possibilities compatible with *q* are less likely than those incompatible with *q*.

#### **Definition 10**

**Rejection of Conjunction.** Let $c=(C,\le )$ be a context.

- $$  c[-p\wedge q]:= {{exec}}({{prop}}^{-q}({{exec}}({{prop}}^{-p}(c))))  $$

**Fig. 4:**
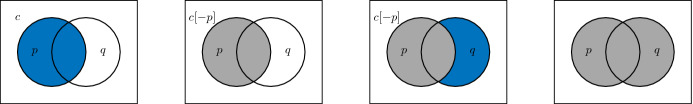
Rejection of conjunction. (1) The *p*-worlds of *c* are less likely than the non-*p*-worlds; (2) context $c[-p]$ is defined (white); (3) the *q*-worlds of $c[-p]$ are less likely than the non-*q*-worlds; (4) the updated context is defined (white)

The final context contains possibilities that are incompatible with *p* and incompatible with *q*. This procedure is shown in Fig. 4.

1. ${{prop}}^{-p}(c)$ is defined: the *p*-worlds in *c* become less likely than the non-*p*-worlds;
2. ${{exec}}({{prop}}^{-p}(c))=c[-p]$ is defined: the *p*-worlds are eliminated;
3. ${{prop}}^{-q}({{exec}}({{prop}}^{-p}(c)))$ is defined: the *q*-worlds in $c[-p]$ become less likely than the non-*q*-worlds;
4. finally, ${{exec}}({{prop}}^{-q}({{exec}}({{prop}}^{-p}(c))))=(c[-p])[-q]$ is defined: the *q*-worlds are eliminated.

Therefore update functions for rejected conjunction and rejected disjunction, $c[-p\wedge q]$ and $c[-p\vee q]$, are truth-conditionally equivalent. Moreover, they deliver the static meaning of *p* *nor* *q*. Rejected conjunction restricts the initial set of possibilities *C* to $(C\backslash \{w:w\in \llbracket p \rrbracket \})\backslash \{w:w\in \llbracket q \rrbracket \}=(C\backslash \llbracket p \rrbracket )\cap (C\backslash \llbracket q \rrbracket )$. Hence, defining the assertion of *p* *nand* *q* as $-p\wedge q$ would not be minimally adequate. Moreover, no combination of the primitives introduced so far (${{prop}}^{+p}, {{prop}}^{+q}$, and ${{exec}}$) is minimally adequate for the assertion of *p* *nand* *q*. Nevertheless the same functions suffice for $\wedge$, $\vee$, and nor. We conjecture that this failure of adequacy is the reason why no natural language has a word *nand.

In this section we have shown how to define minimally adequate rules for *and*, *or*, and *nor*, and that, on the basis of the same primitives, we cannot define rules for *nand. So it goes for material conditional, material bi-conditional, and exclusive disjunction. The only non-trivial connectives definable in the update system correspond to $p\wedge \lnot q$ and $\lnot p\wedge q$. However, these operators fail to be lexicalized if we assume, as we do in related work (Carcassi and Sbardolini, 2022), that mixed polarity increases complexity. For example, the process $(c[+p])[-q]$ upgrades some possibilities and downgrades others, and it is plausible that this lack of homogeneity is costlier (see Geurts and van der Slik (2005) for evidence in support of this claim). For completeness, we note that trivial operators are also definable, verum $\top$ and falsum $\bot$, since some updates are trivial. In this case, it is plausible that we do not observe trivial connectives in the lexicon of natural language for general reasons of economy weighing against semantically inert material. Thus, independently motivated and general cognitive constraints explain the remaining aspects of the lexicalization of connectives.

If the system of bilateral updates we have introduced in this paper characterizes the lexical connectives of natural language, lexical gaps in the Boolean domain are explained by the logic of assertion and rejection, together with general cognitive pressures in favour of simplicity and economy. The explanation rests on assumptions we have made about assertion and rejection, and about the mechanics of update. Of course, other systems could be designed, for example, by postulating new or different primitives. Recall that one of our objections to the Gricean account was one of circularity: its assumed primitives are those of standard logic textbooks or of Boolean algebra, on which *nand contains a negation but *or* does not. The force of this objection rests on the fact that propositional logic and Boolean algebra are standardly presented in a language that includes $\wedge$ and $\vee$ as primitives (or $\sqcap$ and $\sqcup$), but not nand. This presupposes what must be explained. In contrast, the assumptions we have made are motivated by a Stalnakerian conception of speech acts. Update proposals and executions are independently plausible descriptions of the computational processes that operate on an input context. These primitives do not presuppose the absence of nand.

### Negation

It is possible, of course, to express the contradictory of conjunction in natural language, e.g. as *not both* *p* *and* *q*. Moreover, native speakers could learn to use a word expressing this truth-condition if properly taught, so there is nothing inconsistent or incoherent about nand. How can nand be expressed as an update after all?

Complement negation $\sim$ is the negation of Heim and Veltman. $L_{\rm {Bool}}^{\sim }$ is the language $L_{\rm {Bool}}$ extended with complement negation. We assume that $\sim$ is the dynamic contribution of the word *not* (and its analogues in other languages), and that it has its ordinary static semantics: $\llbracket {\sim }\phi \rrbracket =W\backslash \llbracket \phi \rrbracket$. Dynamically too, we follow Heim and Veltman and take positive update by ${\sim }p$ to be the complement of a positive update by *p*. Rejection of complement negation disappears as mere assertion.^9^

#### **Definition 11**

Complement Negation. Let $c=(C,\le )$ be a context and $\phi$ any sentence.

- $$  c[+{\sim }\phi ] := c\backslash c[+\phi ]  $$
- $$  c[-{\sim }\phi ] := c[+\phi ]  $$

Consider the rejection of atomic *p*. As we said, $-p$ restricts the possibilities *C* of a context $(C,\le )$ to $C\backslash \llbracket p \rrbracket$. Asserting ${\sim }p$ has the same informational effect. For $(C,\le )[+{\sim }p]=(C,\le )\backslash (C,\le )[+p]$, whose set of possibilities is $C\backslash (C\cap \llbracket p \rrbracket )=<> <>C\backslash \llbracket p \rrbracket$. It follows that complement negation is minimally adequate.

**Minimal adequacy:** Negation. $(C,\le )[+{\sim }p]=(C\cap \llbracket {\sim }p \rrbracket ,\le )$

Consider updates in $L_{\rm {Bool}}^{\sim }$. The rules for asserted $\wedge ,\vee$, and nor remain as above, and in particular nor is asserted as the rejection of disjunction. Now nand can be asserted as the complement negation of conjunction.

$$  \eqalign{ c[+p\,\textsf { nand }\,q] = c[+{\sim }(p\wedge q)]&= c\backslash c[+p\wedge q] }<!endaligned>  $$

It is obvious that $+p\textsf {\; nand \;}q$, thus defined, is minimally adequate with respect to the static semantics of nand, since the update for asserted conjunction is minimally adequate. However, nand requires an extra step of complexity, because $\sim$ is a relation between contexts.

We need to distinguish two ways to form a negative: by rejection, and by negation properly speaking or complement negation. The two are not the same: rejection is a relation between context and content, governing the type of speech act performed, and negation in a dynamic setting is a relation between contexts (as for Veltman and Heim). Complement negation gives us the four corners of the Aristotelian Square and (though here we omit proofs) full classical logic (see Sbardolini, 2022). It does so by combining compositionally with the connectives that are already lexicalized, just as in English one has to combine *not* with *and* to express *not both p* *and* *q*. However, the expressive resources of the lexicon with regards to the binary connectives include no more than rejection as a means to form a negative. And so the logic of the lexicon only distinguishes between *and, or*, and *nor*. The full expressive resources of natural language include complement negation, and with it the possibility of expressing all Boolean distinctions.

## The quantifiers

A similar explanation applies to the lexicalized quantifiers. Determiners such as English *all, some, no,* are analysed as quantifiers. The lexicalization patterns of determiners and connectives in English are parallel. The Aristotelian Square for quantifiers leaves the **O** corner empty, which is where a word for $\textsf {Nall}$ would be (Horn, 1972). 
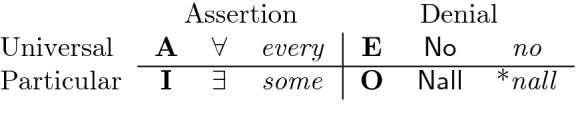


Similar remarks hold for correlated determiners *both, either, neither*, and temporal adverbs *always, sometimes, never*. Though correlated determiners often occur in complex constructions involving the connectives (*both *...*and*...), they may also stand alone (Hendriks, 2004):

**Table:** 

5.	(a) Both hands are needed to clap (b) You may kick the ball with either foot (c) I am deaf in neither ear

It is standard to treat *both, either, neither* as quantifiers carrying a presupposition that their NP restrictor has cardinality 2. The presupposition is satisfied by world knowledge in (5). The treatment of temporal adverbs as quantifiers goes back to Lewis (1975). For these reasons we do not give a separate analysis for temporal adverbs and correlated determiners.

We extend the object language $L_{\rm {Bool}}$ to include the four Aristotelian quantifiers. We add four inductive clauses to the definition of a formula. Let $\phi$ be a formula of the propositional fragment of $L_{\rm {Bool}}$ obtained with the connectives shown above to be minimally adequately assertable without negation ($\wedge ,\vee ,\textsf {nor}$). The language so extended is called *L*.

$$  \eqalign{ \phi :=\ \exists _x\phi \ |\ \forall _x\phi \ |\ \textsf {No}_x\phi \ |\ \textsf {Nall}_x\phi }<!endaligned>  $$

For the static semantics, we proceed in ordinary fashion. There is a non-empty domain of individuals *D*, and we assume for simplicity that it is the same for all worlds, and that predicate extensions are defined at all worlds in the model. We assume standard truth-conditions (which we specify in Definition 12 with abuse of notation, but all of this is familiar).

### **Definition 12**

Static interpretation of the quantifiers

**Table:** 

$\llbracket \forall _x\phi \rrbracket =\{w:D\cap \phi =D\}$	$\qquad \llbracket \textsf {No}_x\phi \rrbracket =\{w:D\cap \phi =\varnothing \}$
$\llbracket \exists _x\phi \rrbracket =\{w:D\cap \phi \not =\varnothing \}$	$\qquad \llbracket \textsf {Nall}_x\phi \rrbracket =\{w:D\cap \phi \not =D\}$

As above, we expect Stalnaker’s Rule to obtain for atomic predication, namely results such as $(C,\le )[+\exists _xP]=(C\cap \llbracket \exists _xP\rrbracket ,\le )$ for *P* an atomic predicate, as a test of minimal adequacy. We proceed essentially as above exploiting the well-known correspondence $\forall /\wedge$ and $\exists /\vee$: universal quantification can be understood as generalized conjunction and existential quantification as generalized disjunction. A collapse on the negative side will be observed, similar to the case of connectives, so that only the assertion of No is adequately definable by the benchmark of Stalnaker’s Rule, and not Nall.

As a technical implementation, we formalize this approach by relativizing an update to a variable assignment *g*. Since we decompose update into two stages, *prop* and *exec*, this technical implementation leaves two options: only *prop* is relative to *g*, or *prop* and *exec* together are relative to *g*. As we will see, this is the difference between $\exists$ and $\forall$: for the former there is a proposal under each assignment and a single execution, for the latter an update is executed for each assignment of an element of the domain to the variable. Thus we generate a local context for each element of the domain in the latter case but not in the former.

This manner of explanation is quite straightforward technically, but to some extent it may appear unsatisfactory, and so we pause here for a cautionary remark. It is not plausible to imagine that when speakers utter a sentence that contains a determiner they “mentally go through” all elements of the domain and calculate for each instance of the quantifier how information carried by the utterance modifies the context. When we say *Every student is asleep* we do not think to ourselves that Alice is asleep, and Bob is asleep, and so forth till we run out of students. We agree that cognitive feasibility should matter in our theory of semantic competence, whether such competence is displayed by a static or a dynamic framework. But the connection between semantics and cognition is indirect at best. After all, the idea that speakers “mentally go through” the possible worlds in their context in order to determine the effect of an update, or even the truth-conditional content of an utterance, is just as wildly implausible. But this is not a reason to reject possible world semantics. In a similar spirit, we offer a description of the constraints imposed on the transfer of information by utterances with a certain force and content, as they are manifested in the lexicon. The steps required to calculate an update under these constraints need not be those of whatever algorithm speakers run in their cognitive systems.

### Universal and existential

A positive update by an existential $\exists _xP$ should intuitively change the input context so that the worlds surviving the update are all and only those in which something is *P*. In the update rule for asserted existential quantifier, a proposal is made for each variable assignment, and these proposals are executed together. Intuitively, if $\exists _xP$ is asserted, a world *w* in the input context *c* is at least as likely as another *v* if *a* is *P* at *w* but not at *v*, or if *b* is *P* at *w* but not at *v*, and so on for all elements of the domain. The less likely worlds are then eliminated in a single update restriction. We indicate this in our notation by indexing the variable assignment *g* to the proposal, and thus within the scope of the execution operator ${{exec}}$. For technical purposes, we assume that the elements of a domain *D* are linearly ordered beginning with element *i*, the next being element $i+1$, and so on. As a notational convention, we write ‘${{prop}}^{+P_x}_{g(n)}(c)$’ to indicate in compressed form the full expansion ${{prop}}^{+P_x}_{g(x/i)}( {{prop}}^{+P_x}_{g(x/i+1)}(\ldots (c))$, for all the *n* elements of *D*.

#### **Definition 13**

**Assertion of Existential.** Let $c=(C,\le )$ be a context, *D* a domain of individuals, and *g* a variable assignment.

- $$  c[+\exists _xP]:= {{exec}}( {{prop}}^{+P_x}_{g(n)}(c))  $$

**Fig. 5:**
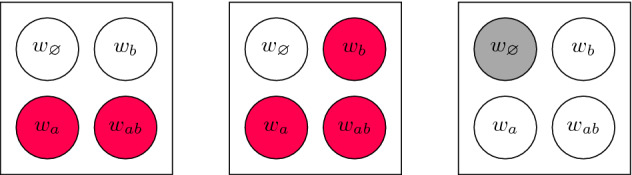
Assertion of Existential. (1) The worlds of *c* compatible with $P_a$ become at least as likely as those incompatible with it (red); (2) the worlds of *c* compatible with $P_b$ become at least as likely as those incompatible with it; (3) the update is executed and the less likely worlds are eliminated

Consider a simple two-elements model with $D=\{a,b\}$, as in Fig. 5. There we only consider four worlds, $w_{\varnothing }, w_a, w_b, w_{ab}$: the extension of *P* is empty in $w_{\varnothing }$, it includes only *a* in $w_a$, and so on. The procedure $+\exists _xP$ is then in three steps.

1. ${{prop}}^{+P_x}_{g(x/a)}(c)$ is defined: all worlds of *c* in which *a* is *P* become at least as likely as those in which it isn’t. Thus $w_a$ and $w_{ab}$ are minimal according to this first proposal.
2. ${{prop}}^{+P_x}_{g(x/b)}({{prop}}^{+P_x}_{g(x/a)}(c))$ is defined: the worlds of *c* in which *b* is *P* become at least as likely as those in which it isn’t. Thus, $w_b$ is added to the minimal worlds.
3. ${{exec}}( {{prop}}^{+P_x}_{g(x/b)}( {{prop}}^{+P_x}_{g(x/a)}(c)))$ is defined: the less likely worlds are eliminated. This results in $\{w_a,w_b,w_{ab}\}$.

It follows that a world survives the update just in case some individual in the domain of the model is *P* at that world, not necessarily the same individual for all worlds. Therefore, if *C* is the information of an initial context *c*, the result of $c[+\exists _xP]$ is to restrict *C* to $\{w\in C:D\cap P\not =\varnothing \}$. Hence the rule for asserting an existential is minimally adequate.

**Minimal adequacy:** Existential. $(C,\le )[+\exists _xP]=(C\cap \llbracket \exists _xP \rrbracket ,\le )$

Next we consider the assertion of a universal. Intuitively, we take $\forall$ to be a generalized conjunction, namely a sequence of updates, one for each assignment of an element of the domain to the quantified variable, with each update applying to the local context defined by the previous. While in asserting an existential the execution ${{exec}}$ is applied only once for all elements of the domain, in asserting a universal each element determines an application of ${{exec}}$. We indicate this in the notation by placing the index of the variable assignment on ${{exec}}$. Thus if $\forall _xP$ is asserted, for an assignment of *a* to *x*, worlds incompatible with $P_a$ are deemed less likely to be actual than worlds compatible with $P_a$, and the update is executed. This is then repeated for all elements of *D*. By ‘${{exec}}_{g(n)}(c)$’ we indicate the full expansion ${{exec}}( {{prop}}^{+P_x}_{g(x/i)}( {{exec}}( {{prop}}^{+P_x}_{g(x/i+1)}(\ldots (c))$ for all the *n* elements of *D*.

#### **Definition 14**

**Assertion of Universal.** Let $c=(C,\le )$ be a context, *D* a domain of individuals, and *g* a variable assignment.

- $$  c[+\forall _xP]:={{exec}}_{g(n)}({{prop}}^{+P_x}(c))  $$

**Fig. 6:**
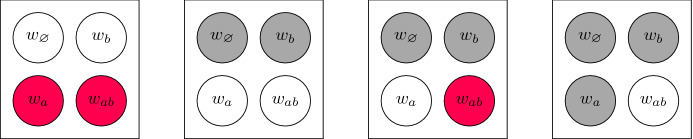
Assertion of Universal. (1) The worlds of *c* compatible with $P_a$ become at least as likely as those incompatible with it; (2) the update is executed; (3) the surviving worlds compatible with $P_b$ become at least as likely as the remaining ones; (4) the second update is executed

The process is depicted in Fig. 6 for the simple two-elements model. At the end, only world $w_{ab}$ survives.

1. ${{prop}}^{+P_x}_{g(x/a)}(c)$ is defined: the worlds of *c* in which *a* is *P* become at least as likely as those in which it isn’t. This puts $w_a$ and $w_{ab}$ among the minimal worlds.
2. ${{exec}}( {{prop}}^{+P_x}_{g(x/a)}(c))$ is defined: the less likely worlds are eliminated, namely $w_{\varnothing }$ and $w_b$, in which the extension of *P* does not include *a*. So far, the result is equivalent to $c[+P_a]$, which is how we may indicate the local context just defined.
3. ${{prop}}^{+P_x}_{g(x/b)}( {{exec}}( {{prop}}^{+P_x}_{g(x/a)}(c)))$ is defined: the worlds of the input context in which *b* is *P* become at least as likely as those in which it isn’t. This puts $w_{ab}$ among the minimal worlds.
4. ${{exec}}({{prop}}^{+P_x}_{g(x/b)}( {{exec}}( {{prop}}^{+P_x}_{g(x/a)}(c))))$ is defined: the less likely worlds are eliminated, namely $w_a$, in which the extension of *P* does not include *b*.

The only possibility left is $w_{ab}$, in which everything is *P*. Consequently, if *C* is the information of the initial context, the effect of asserting $\forall _xP$ is to restrict it to $\{w\in C:D\cap P=D\}$, and so the assertion rule for the universal is adequate.

**Minimal adequacy:** Universal. $(C,\le )[+\forall _xP]=(C\cap \llbracket \forall _xP \rrbracket ,\le )$

As in the previous section, a natural way to obtain negative updates is by converting assertions into rejections. This will give us No but not Nall.

### Negative quantifiers

Rejected existential and rejected universal collapse on the same truth-conditions, which is adequate for the assertion of No. The rules below are obtained by downgrading worlds that in the corresponding positive update were upgraded. Consider the existential first.

#### **Definition 15**

**Rejection of Existential.** Let $c=(C,\le )$ be a context, *D* a domain of individuals, and *g* a variable assignment.

- $$  c[-\exists _xP]:= {{exec}}( {{prop}}^{-P_x}_{g(n)}(c))  $$

**Fig. 7:**
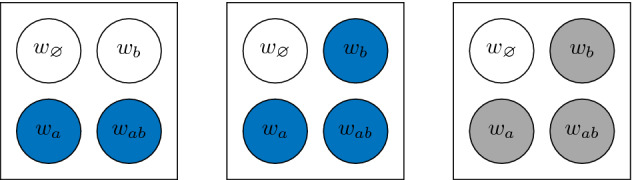
Rejection of Existential. (1) The worlds of *c* compatible with $P_a$ become less likely than those incompatible with it; (2) the worlds of *c* compatible with $P_b$ become less likely than those incompatible with it; (3) the update is executed and the less likely worlds are eliminated

For the rejection of an existential, we go through the domain *D*, and assign low likelihood to worlds in which an element of *D* belongs to *P*. The updated context will contain only the minimal worlds, hence no such worlds. Figure 7 represents this procedure for the two-elements model, the result of which, $\{w_{\varnothing }\}$, is straightforwardly seen to be the complement of a positive update by the existential. Thus, for *C* the information of the initial context, the effect of rejecting an existential is $\{w\in C:D\cap P=\varnothing \}$. We make the reasonable hypothesis that an assertion of $\textsf {No}_xP$ is tantamount to a rejection of $\exists _xP$, and *vice versa*.

#### **Definition 16**

**Assertion and Rejection of No.** Let $c=(C,\le )$ be a context.

- $$  c[+\textsf {No}_xP]:=c[-\exists _xP]  $$
- $$  c[-\textsf {No}_xP]:= c[+\exists _xP]  $$

From the reasoning above, No is minimally adequate.

**Minimal adequacy:** No. $(C,\le )[+\textsf {No}_xP]=(C\cap \llbracket \textsf {No}_xP \rrbracket ,\le )$

**Fig. 8:**
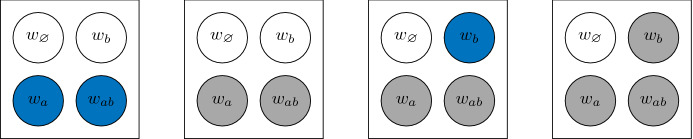
Rejection of Universal. (1) The worlds of *c* compatible with $P_a$ become less likely than those incompatible with it; (2) the update is executed; (3) of the surviving worlds, those compatible with $P_b$ become less likely than the remaining ones; (4) the second update is executed

Finally, we turn to universal rejection. This is a sequence of updates in which at each step worlds where something is *P* are assigned low likelihood. A local context is defined after each step.

#### **Definition 17**

**Rejection of Universal.** Let $c=(C,\le )$ be a context, *D* a domain of individuals, and *g* a variable assignment.

- $$  c[-\forall _xP]:=\text {\it{exec}}_{g(n)}(\text {\it{prop}}^{-P_x}(c))  $$

With respect to Fig. 8, consider *a* first. Worlds in which *a* is *P* are given low likelihood, hence $w_a$ and $w_{ab}$. Then they are eliminated by the execution of the update. There remains a local context with respect to which $P_b$-worlds are given low likelihood. The execution keeps only the minimal worlds, hence the non-$P_b$-worlds, hence only $w_{\varnothing }$.

Therefore, $c[-\forall _x\phi ]=c[-\exists _x\phi ]$. But a condition of minimal adequacy based on Stalnaker’s Rule would have that updating a set of worlds *C* with the **O** corner quantifier $\textsf {Nall}_xP$ should deliver $\{w\in C:D\cap P\not =D\}$. Hence Nall cannot be defined as a rejected universal. Inspection of the rules and of the primitives admitted so far shows that the bilateral framework does not include an adequate rule for Nall. As we conjectured above, if the logic of the lexicon in natural language is captured by the bilateral updates we have introduced, failure of minimal adequacy explains why Nall is missing.

However, classical logic is just behind the corner. We may extend *L* to $L^{\sim }$, on the assumption that lexicalized negation *not* is complement negation, as above. Then we can calculate the assertion of $\textsf{Nall}_xP$ by $c[+{\sim }\forall _xP]=c\backslash c[+\forall _xP]$. As above, we remark that negation adds an extra cognitive step that goes beyond the procedures described only in terms of assertion and rejection on a single context *c*, and that characterize the lexical material: the added expressive resources crucially allow to manipulate two contexts at once, and to define all Aristotelian quantifiers.

This completes our account of the update potentials of familiar logical operators. All remaining Boolean connectives and Boolean combinations of the operators definable in the bilateral system can be introduced from here, following essentially Keenan and Stavi (1986). We have extended standard update semantics in two directions: first, by allowing for rejection alongside assertion; second, by allowing sentences to perform an update collectively rather than just one by one. Both innovations are made possible by analysing update as a complex operation comprising two stages: proposal (which can be positive or negative) and execution (which need follow at least one proposal, possibly more). These generalizations let us define classical meanings for $\wedge ,\vee ,\exists ,\forall$, and also nor and No, but not for nand and Nall. This gives us a logical reason for the asymmetry observed in natural language within the Aristotelian Square.

There is an added advantage to our account. The monotonicity properties of lexicalized connectives and determiners are predicted to follow. Intuitively, an operator is upward monotone in an argument position that is under $+$, and downward monotone under −. Thus upward and downward monotonicity are not just assumed but follow from the logic of assertion and rejection.

## Monotonicity

The monotonicity universal states that all natural language lexical determiners are monotone (Barwise and Cooper, 1981). Let $\phi ,\psi$ be sets of individuals, with $\phi \subseteq \psi$.

$$  \eqalign{ \hbox{ A } \hbox{ quantifier } \textsf {Q} \hbox{ is } \hbox{ upward } \hbox{ monotone }&\hbox{ iff }{\textsf {Q}_x\phi\, \vDash\, \textsf {Q}_x\psi }\cr \hbox{ downward } \hbox{ monotone }&\hbox{ iff }{\textsf {Q}_x\psi\, \vDash\, \textsf {Q}_x\phi } }<!endaligned>  $$

The system of update potentials we presented defines only monotone operators. Update itself is a restriction of the initial context according to our definitions. Moreover, the polarity of the update determines the direction of monotonicity: positive updates are upward monotone, negative updates are downward monotone.

We begin by generalizing the notion of monotonicity to arbitrary types, following Peters and Westerståhl (2006). As usually defined, validity $\vDash$ is the subset relation in the model theory, thus the metalinguistic statement $\phi\, \vDash\, \psi$ may be understood as $\llbracket \phi \rrbracket \subseteq \llbracket \psi \rrbracket$. For readability, we omit the interpretation function from the following. Let *e* be the type of individuals, *s* the intensional type, and $\alpha$ a metavariable over variables for types.

### **Definition 18**

Monotonicity for arbitrary type

Let $A,A'$ be sets of type $\alpha t$ and $A\subseteq A'$. A one-place operator Q is:

mon $$  \uparrow  $$iff $$  \textsf {Q}A\subseteq \textsf {Q}A'  $$

mon $$  \downarrow  $$iff $$  \textsf {Q}A'\subseteq \textsf {Q}A  $$

This definition entails that the existential quantifier is upward monotone mon $\uparrow$, and No is mon $\downarrow$. Next, we generalize to *n*-ary operators.

### **Definition 19**

Monotonicity for arbitrary type and arbitrary number of arguments

Let $A_1,\ldots ,A_n$ be sets of type $\alpha t$ and $A_i\subseteq A'_i$ for $1\le i\le n$. An *n*-place operator Q is:

mon $\uparrow$in its *i*-th argument iff $\textsf {Q}(A_1,\ldots ,A_i,\ldots ,A_n)\subseteq \textsf {Q}(A_1,\ldots ,A'_i,\ldots ,A_n)$

mon $\downarrow$in its *i*-th argument iff $\textsf {Q}(A_1,\ldots ,A'_i,\ldots ,A_n)\subseteq \textsf {Q}(A_1,\ldots ,A_i,\ldots ,A_n)$

We call an operator mon $\uparrow$(mon $\downarrow$) *simpliciter* just in case it is mon $\uparrow$ (mon $\downarrow$) in all its arguments. It is easy to verify that $\wedge$ and $\vee$ are mon $\uparrow$ and nor is mon $\downarrow$ in this general sense, since the following statements hold by their static semantics and their converses fail (Humberstone, 2011, p. 490):

$$  \eqalign{ \phi \wedge \phi '&\subseteq (\phi \vee \psi )\wedge \phi '\cr \phi \vee \phi '&\subseteq (\phi \vee \psi )\vee \phi '\cr (\phi \vee \psi )\,\textsf { nor }\,\phi '&\subseteq \phi\, \textsf { nor }\, \phi ' }<!endaligned>  $$

These logical connections are observable in natural language. For instance, (6a) entails (6b) while $\llbracket$ lifted a finger $\rrbracket \subseteq \llbracket$ moved $\rrbracket$, and not *vice versa*.

**Table:** 

6.	(a) Julia hasn’t moved nor spoken since yesterday (b) Julia hasn’t lifted a finger nor spoken since yesterday

We now divide the definable operators in two groups: those in whose proposal worlds are upgraded and those in whose proposal worlds are downgraded. Let ${\mathcal {A}}=\{\wedge ,\vee ,\exists ,\forall \}$ be the former group and A any operator in it, and let ${\mathcal {R}}=\{\textsf {nor},\textsf {No}\}$ be the latter group and R any operator in it. The following result holds.

- **Theorem 1.** Monotonicity of Update. Let $A_1,\ldots ,A_n$ be sets of type $\alpha t$ and $A_i\subseteq A'_i$ for $1\le i\le n$.
  $$  \eqalign{ c[+\textsf {A}(A_1,\ldots ,A_i,\ldots ,A_n)]&\subseteq c[+\textsf {A}(A_1,\ldots ,A'_i,\ldots ,A_n)]\cr c[+\textsf {R}(A_1,\ldots ,A'_i,\ldots ,A_n)]&\subseteq c[+\textsf {R}(A_1,\ldots ,A_i,\ldots ,A_n)] }<!endaligned>  $$

### *Proof*

Consider A operators first. We write the connectives with prefix notation. Consider the functions from context to context $[+{\wedge }(A_1,\ldots ,A_n)]$, $[+{\vee }(A_1,\ldots ,A_n)]$, $[+\exists _xA_i]$ and $[+\forall _xA_i]$. Since application of these functions results in minimally adequate updates, it follows that for any *i*, $1\le i\le n$, if $A_i\subseteq A'_i$, then $c[+{\wedge }(A_1,\ldots , A_i,\ldots ,A_n)]\subseteq c[+{\wedge }(A_1,\ldots ,A'_i,\ldots ,A_n)]$. As for R operators, applications $[+\textsf {nor}(A_1,\ldots ,A_i,\ldots ,A_n)]$ and $[+\textsf {No}_xA_i]$ results in minimally adequate updates. It follows that if $A_i\subseteq A'_i$, $c[+\textsf {No}_xA_i']\subseteq c[+\textsf {No}_xA_i]$. Likewise for the remaining operators in this group.$\square$

The next generalization is an immediate consequence.

- **Theorem 2.** Monotonicity of Minimally Adequately Assertable Logical Operators. Let $A_1,\ldots ,A_n$ be sets of type $\alpha t$ for $1\le i\le n$. If $\textsf {Q}\in {\mathcal {A}}\cup {\mathcal {R}}$, then $c[+\textsf {Q}(A_1,\ldots ,A_n)]$ is monotone.

We call an operator mon $\uparrow$ (mon $\downarrow$) just in case its corresponding update is mon $\uparrow$ (mon $\downarrow$) in the sense above. Then, A operators $(\{\wedge ,\vee ,\exists ,\forall \})$ are mon $\uparrow$ and R operators $(\{\textsf {nor},\textsf {No}\})$ are mon $\downarrow$. So, all lexicalized operators are monotone.

Beginning with Barwise and Cooper (1981), it has been observed that lexicalized quantifiers are monotone. Lexicalized connectives are similarly monotone. It is also known that there are monotone operators that aren’t realized in the lexicon of any natural language. The present account suggests a possible explanation for the underlying universal: monotonicity is a by-product of the accumulation of information in conversation, given a basic distinction between assertion and rejection. The explanation is direct, and based on the logic of natural language expressions, as they are encoded by bilateral updates.

## Conclusion

This paper is about the lexicalizability of logical operators in natural language. We began by criticizing a Gricean account due to Horn (1989) and recently discussed by Katzir and Singh (2013) and Uegaki (2022). We raised two concerns: first, the Gricean account relies on an arbitrarily curtailed notion of ‘covering’; second, the primitives of the Gricean account implicitly assume what is supposed to be explained. As an alternative, we presented a logic-based account of lexicalizability. The logic of assertion and rejection in context breaks the symmetry enforced by classical reasoning on the Aristotelian Square of Oppositions.

The primitives of our account are justified as basic operational steps in a conception of updates as procedures to change an input context. The lexicon does not distinguish binary Boolean connectives beyond *and, or*, and *nor*. Neither does the logic of bilateral updates we have introduced. Classical logic, and all its distinctions, can be recaptured with the help of complement negation, i.e. the operator expressed by overt negation *not*. This is how all classical meanings are typically expressed in natural language as well.

There are several directions for further research. An immediate concern is to extend the bilateral dynamic approach to lexical gaps in other areas of the lexicon. Our discussion left out of the picture modals (*might, must*) and proportional quantifiers (*most, few*), among others. An account should be given of the presence of quantificational focus-sensitive operators such as *even* and *only*, and the absence of alternative operators of the same type. Similar questions could be raised concerning conditionals (*if*, *unless*). All these questions presuppose that the semantics of these expressions is understood at least reasonably well. Another direction for further work is to integrate the present model of discourse with other speech acts such as questions and imperatives, which we have not discussed. Some recent work, particularly Farkas and Bruce (2010) and Murray and Starr (2021), seem at least compatible with our approach and conceptually close, but many details should be fleshed out.

## Appendix: Compositionality and Veltman’s disjunction

In this Appendix we discuss formal properties of update functions for the propositional fragment of the language $L_{\rm {Bool}}$, including compositional definitions thereof. We will also compare the bilateral update system with classical update semantics systems.

On our treatment, disjunction determines an update executed once, which incorporates information from all disjuncts. In order to state an update function for disjunction recusively in the traditional ‘$c[\cdot ]$’ notation, we introduce the following convention: the argument of ‘$c[\cdot ]$’ is at least one but possibly more sentences. The sentences enclosed within brackets $[\cdot ]$, together with their force sign $+$ or −, determine what proposal is made. We can then define update functions recursively. Let $+\Gamma =\{+p_1,+p_2,\ldots \}$ and $-\Gamma =\{-p_1,-p_2,\ldots \}$ for $p_1,p_2,\ldots$ atomic sentences.

$$  \eqalign{ c[+\Gamma ]&=c\cap \{w\in \llbracket p_i \rrbracket :{+p_i}\in {+\Gamma }\}\cr c[-\Gamma ]&=c\backslash \{w\in \llbracket p_i \rrbracket :{-p_i}\in {-\Gamma }\} }<!endaligned>  $$

Updates by a single sentence are just special cases of this.

$$  \eqalign{ c[+p]&= c\cap \{w\in \llbracket p \rrbracket \}\cr c[-p]&= c\backslash \{w\in \llbracket p \rrbracket \} }<!endaligned>  $$

Updates are then compositionally extended to all adequately definable operators as follows (where any occurrence of $\Gamma$ may be empty).

**Table:**

$$ c[\Gamma ]=c[+\Gamma ]\cup c[-\Gamma ] $$	
$c[+\phi \wedge \psi ,\Gamma ]=c[+\phi ][+\psi ]\cup c[\Gamma ]$	$c[-\phi \wedge \psi ,\Gamma ]=c[-\phi ][-\psi ]\cup c[\Gamma ]$
$c[+\phi \vee \psi ,\Gamma ]=c[+\phi ,+\psi ,\Gamma ]$	$c[-\phi \vee \psi ,\Gamma ]=c[-\phi ,-\psi ,\Gamma ]$
$c[+\phi \textsf {\; nor \;}\psi ,\Gamma ]=c[-\phi ,-\psi ,\Gamma ]$	$c[-\phi \textsf {\; nor \;}\psi ,\Gamma ]=c[+\phi ,+\psi ,\Gamma ]$

Update by a set of assertions and rejections is the union of updates by assertions and by rejections, conjunction is a sequence of updates, and disjunction is eliminated within the square brackets. In general, set theoretic union is used to model the comma inside square brackets. In this system, conjunction is symmetric: $\phi \wedge \psi$ if and only if $\psi \wedge \phi$. Indeed, the bilateral update semantic discussed in this paper is ‘strongly static’ in the sense of Rothschild and Yalcin (2016, p. 340). This is because there aren’t “genuinely dynamic” expressions in the language we are considering: no anaphoric expressions, modals, or conditionals. Such genuinely dynamic expressions are necessary in order for the asymmetry of conjunction to become visible. Extensions of the present framework to deal with anaphora and other dynamic phenomena are left for a separate occasion.

We finally turn to disjunction. Several rules for disjunction have been proposed in the literature on dynamic semantics. All proposals are truth-conditionally equivalent but make slightly different predictions concerning presupposition projection. The following are some prominent candidates.

$$  \eqalign{ c[\phi \vee \psi ]&= c[\phi ]\cup (c\backslash c[\phi ])[\psi ]\cr c[\phi \vee \psi ]&= (c\backslash c[\psi ])[\phi ]\cup c[\psi ]\cr c[\phi \vee \psi ]&= c[\phi ]\cup c[\psi ] }<!endaligned>  $$

The first option is due to Heim (1983, 1990), also endorsed by Rothschild (2011). The second option is mentioned by Rothschild (2011). The third option is mentioned by Soames (1989), and endorsed by Veltman (1996); Simons (2000, 2001), and in inquisitive semantics (Ciardelli et al., 2018; Roelofsen and Dotlačil, 2019). As we did not discuss presupposition projection, we do not take a stand on these debates. Our limited aim in this Appendix is to clarify the relation between the rule for disjunction we introduced, and these various options.

Of course, the candidate rules for disjunction stated above are all to be understood as conditions for the assertion of disjunction. In a bilateral framework the condition on the left of the identity sign would be $c[+\phi \vee \psi ]$, and the remaining updates are also to be understood as assertoric. A more important observation is that the first and second rule make use of complement negation, and so implicitly assume that disjunction “contains” a negation, or at least that disjunction presupposes complement negation. This is an incautious assumption to make when we are trying to be careful about the conceptual primitives of a semantic system. We ought to be careful about the primitives in order to explain what can be lexalized. If the rule for disjunction is captured by the first or second option, a semantic system that can express disjunction at lexical level would appear to have the resources to express nand at lexical level too.

There remains Veltman’s rule. Indeed, the disjunction rule we have proposed simplifies to Veltman disjunction in the ‘positive’ (assertoric) fragment of $L_{\rm {Bool}}$. Consider $c[+\phi \vee \psi ]=c[+\phi ,+\psi ]$. Suppose that $\phi$ and $\psi$ do not contain an occurrence of negation. Then we reason by induction on syntactic complexity (here, we sketch the argument). For the base of induction, suppose that $\phi :=p$ and $\psi :=q$, and let $c=(C,\le )$. Then $(C,\le )[+p,+q]=(C\cap \{w\in \llbracket p \rrbracket \cup \llbracket q \rrbracket \},\le )$. Then *C* reduces to $C\cap (\llbracket p \rrbracket \cup \llbracket q \rrbracket )$. Since intersection distributes over union, the result is equivalent to $(C\cap \llbracket p \rrbracket )\cup (C\cap \llbracket q \rrbracket )$, which is an instance of Veltman’s rule. If $\phi$ is a disjunction, the disjunction is eliminated inside the square brackets. If $\phi$ is a conjunction, the rule for conjunction is applied to $c[+\phi _1\wedge \phi _2,+\psi ]$ and Veltman’s rule is obtained by the induction hypothesis through $c[+\phi _1][+\phi _2]\cup c[+\psi ]$. The same holds of the second argument $\psi$. In the presence of rejection, Veltman’s rule is no longer equivalent with ours. This is because Veltman’s rule behaves classically: $c[-p] \cup {c}[-q]$ delivers the truth-conditions of nand, but of course at the cost of taking the input context twice in two updates computed in parallel and then disjoined. Without rejection the two rules are indistinguishable. We leave a more detailed comparison of the present system with different dynamic systems for another occasion.

## Footnotes

1 As the notation indicates, we are simplifying. Natural language determiners are usually analysed as generalized quantifiers, and for such two-argument structures a distinction is made between monotonicity in scope position and monotonicity in restriction position. The relevant notion for present purposes is the former: although we will not discuss proportional quantifiers, it is only monotonicity in scope position that could be a universal, since the quantifier *most* is not monotonic in its restriction argument. Moreover, our discussion is not affected by the simpler conventions of standard predicate logic, and so we discuss “ungeneralized” quantifiers throughout. See Peters and Westerståhl (2006) for a thorough presentation of quantification theory and natural language determiners.

2 Other universals have been proposed besides monotonicity, such as conservativity (Barwise and Cooper, 1981; von Fintel and Keenan, 2019) and connectedness (Chemla et al., 2019). However, Nall is straightforwardly conservative and connected, and so these properties do not explain the absence of **nall*.

3 In effect, our proposal is to ‘triangularize’ the Aristotelian Square. There is a tradition of attempts to triangularize the square discussed in Horn (2012), which however has little in common with our approach. In this tradition, *or* and *nand* are made to coincide (as well as *some* and *nall*), because *or* is taken to mean exclusive disjunction *either and not both* (and *some* to mean *some and not all*). In contrast, on our approach the lexical connectives have their classical meanings, but the square is triangulated at speech act level. See Sbardolini (2022) for more discussion of this point.

4 Some languages, such as Maricopa (Gil, 1991) and ASL (Davidson, 2013) lexicalize less than the full set of operators of a language like English. Our analysis is about lexicalizability in principle. Horn-style pressures to lexicalize less than what’s naturally possible are well motivated, and may induce languages to develop specific lexicalization strategies. Additional considerations perhaps of this kind may explain why in some cases **E** operators are absent. See Carcassi and Sbardolini (2022) for an account of cross-linguistic lexicalization patterns based on the present approach.

5 As a reviewer points out, a natural way of stating the constraint on complexity we are assuming is this: updates that minimize complexity are those that can be expressed as functions of a single context. Additional inputs increase complexity.

6 That being said, this paper does not include an extensive discussion on how to integrate its conclusions with existing literature on disjunction and alternatives. It may be that some of the competing views in the literature are closer to our approach than others, but this is something we leave for future work.

7 For simplicity, we set aside presupposition failures and inconsistencies in the update dynamics: all worlds are either $\phi$ or not-$\phi$ for any $\phi$, no world is both, and interlocutors are equally informed and equally opinionated concerning which worlds are more likely to be the actual world.

8 Therefore, adding the information of multiple sentences does not turn the pre-order $\le$ into a linear order: the $p\wedge q$-worlds are not uniquely “best” in the ordering. Consider a context $\{w_{pq}, w_p, w_q, w_{\varnothing }\}$, with subscripts indicating which sentences are true at a world. Assuming all worlds are initially equally likely, the step ${{prop}}^{+p}(c)$ places all *p*-worlds at least as low as the non-*p*-worlds and everything else stays the same: $\{w_{pq},w_p\}\le \{w_q,w_{\varnothing }\}$. The step ${{prop}}^{+q}({{prop}}^{+p}(c))$ places all *q*-worlds at least as low as the non-*q*-worlds and everything else stays the same: $\{w_{pq},w_p,w_q\}\le \{w_{\varnothing }\}$. The final step *exec* eliminates the least likely world left.

9 A technicality: a context *c* is not a set but an ordered pair, and set-theoretic subtraction $\backslash$ is not defined on ordered pairs. Let $(C,\le )$ and $(C',\le ')$ be contexts. We define $(C,\le )\backslash \backslash (C',\le '):=(C\backslash C',{\le }|^{C\backslash C'})$. That is, subtracting a context $(C',\le ')$ from a context $(C,\le )$ involves removing the possibilities of the second from the first, and restricting its order to the possibilities that are left. When the backslash $\backslash$ occurs between contexts in the main text, consider it a systematic typo: it’s really two backslashes $\backslash \backslash$, defined as we just did.

## References

[CR1] Alonso-Ovalle, L. (2006). *Disjunction in alternative semantics*. Ph. D. thesis, University of Massachussetts at Amherst.

[CR2] Barwise J, Cooper R (1981). Generalized quantifiers and natural language. Linguistics and Philosophy.

[CR3] Berto F, Restall G (2019). Negation on the Australian Plan. Journal of Philosophical Logic.

[CR4] Carcassi, F. & Sbardolini G. (2022). Assertion, rejection, and the evolution of Boolean operators. Ms., University of Amsterdam.

[CR5] Chemla E, Buccola B, Dautriche I (2019). Connecting content and logical words. Journal of Semantics.

[CR6] Ciardelli I, Groenendijk J, Roelofsen F (2018). Inquisitive Semantics.

[CR7] Davidson K (2013). ‘And’ or ‘or’: General use coordination in ASL. Semantics and Pragmatics.

[CR8] De Swart H, von Heusinger K, Egli U (2000). Scope ambiguities with negative quantifiers. Reference and Anaphoric Relations.

[CR9] De Swart H (2010). Expression and Interpretation of Negation: An OT Typology.

[CR10] Dimroth C, Horn L (2010). The acquisition of negation. The expression of negation.

[CR11] Farkas DF, Bruce KB (2010). On reacting to assertions and polar questions. Journal of Semantics.

[CR12] Frana I, Rawlins K (2019). Attitudes in discourse: Italian polar questions and the particle mica. Semantics and Pragmatics.

[CR13] Geurts B (1996). On no. Journal of Semantics.

[CR14] Geurts B, van der Slik F (2005). Monotonicity and Processing Load. Journal of Semantics.

[CR15] Gil D, Zaefferer D (1991). Aristotle goes to arizona and finds a language without ‘and’. Semantic Universals and Universal Semantics.

[CR16] Ginzburg J, Seligman J (1996). Dynamics and the semantics of dialogue. Language, Logic, and Computation.

[CR17] Groenendijk, J. & Stokhof M. (1984). *Studies on the semantics of questions and the pragmatics of answers*. Ph. D. thesis, University of Amsterdam.

[CR18] Heim, I. (1983). On the projection problem for presuppositions. Reprinted in P. Portner & B. H. Partee (Eds.), *Formal semantics—the essential readings* (pp. 249–260). Oxford: Blackwell, 2002.

[CR19] Heim, I. (1990). Presupposition projection. In R. van der Sandt (Ed.), *Reader for the Nijmegen Workshop on Presupposition, Lexical Meaning, and Discourse Processes.* University of Nijmegen.

[CR20] Hendriks P, ter Meulen A, Abraham W (2004). Either, both, and neither in coordinate structures. The Composition of Meaning: From Lexeme to Discourse.

[CR21] Higginbotham J, May R (1981). Questions, quantifiers and crossing. Linguistic Review.

[CR22] Horn, L. (1972). *On the semantic properties of the logical operators in English*. Ph. D. thesis, UCLA.

[CR23] Horn, L. (2012). Histoire d’*o: Lexical pragmatics and the geometry of opposition. In J.-Y. Beziau & G. Payette (Eds.), *The square of opposition. A general framework for cognition* (pp. 393—426). Bern: Peter Lang.

[CR24] Horn L (1989). A Natural History of Negation.

[CR25] Humberstone L (2011). The Connectives.

[CR26] Incurvati L, Schlöder JJ (2017). Weak Rejection. Australasian Journal of Philosophy.

[CR27] Jespersen O (1917). Negation in English and other languages.

[CR28] Katzir R, Singh R (2013). Constraints on the lexicalization of logical operators. Linguistics and Philosophy.

[CR29] Keenan EL, Faltz LM (1986). Boolean Semantics for Natural Language.

[CR30] Keenan EL, Stavi J (1986). A semantic characterization of natural language determiners. Linguistics and Philosophy.

[CR31] Landman F (2004). Indefinites and the type of sets.

[CR32] Lewis, D. K. (1975). Adverbs of quantification. In E. L. Keenan (Ed.), *Formal semantics of natural language* (pp. 178–188). Cambridge: Cambridge University Press.

[CR33] Murray SE, Starr WB (2021). The structure of communicative acts. Linguistics and Philosophy.

[CR34] Peters S, Westerståhl D (2006). Quantifiers in Language and Logic.

[CR35] Price H (1990). Why ‘not’?. Mind.

[CR36] Roelofsen, F., & Dotlačil, J. (2019). Dynamic inquisitive semantics: Anaphora and questions. In M. T. Espinal, E. Castroviejo, M. Leonetti, L. McNally, & C. Real-Puigdollers (Eds.), *Proceedings of Sinn und Bedeutung*,* 23*(1), 365–382.

[CR37] Rothschild D (2011). Explaining presupposition projection with dynamic semantics. Semantics and Pragmatics.

[CR38] Rothschild D, Yalcin S (2016). Three notions of dynamicness in language. Linguistics and Philosophy.

[CR39] Rumfitt I (2000). ‘Yes’ and ‘no’. Mind.

[CR40] Sauerland, U. (2000). No ‘no’: On the crosslinguistic absence of a determiner ‘no’. In *Proceedings of the Tsukuba workshop on determiners and quantification* (pp. 415–444). Tsukuba: Tsukuba University.

[CR41] Sbardolini, G. (2022). The logic of lexical connectives. Ms., University of Amsterdam.

[CR42] Schlenker, P. (2009). Local contexts. *Semantics and Pragmatics,**2,* 1–79.

[CR43] Simons M (2000). Issues in the Semantics and Pragmatics of Disjunction.

[CR44] Simons M (2001). Disjunction and alternativeness. Linguistics and Philosophy.

[CR45] Smiley, T. (1996). Rejection. *Analysis,**56,* 1–9.

[CR46] Soames S, Gabbay D, Guenthner F (1989). Presupposition. Handbook of Philosophical Logic.

[CR47] Stalnaker R (1978). Assertion. Syntax and Semantics.

[CR48] Steinert-Threlkeld S, Szymanik J (2019). Learnability and semantic universals. Semantics and Pragmatics.

[CR49] Steinert-Threlkeld S, Szymanik J (2020). Ease of learning explains semantic universals. Cognition.

[CR50] Uegaki W (2022). The informativeness/complexity trade-off in the domain of Boolean connectives. Linguistic Inquiry.

[CR51] van Benthem J (1984). Questions about quantifiers. Journal of Symbolic Logic.

[CR52] van Benthem J (2007). Dynamic logic for belief revision. Journal of Applied Non-Classical Logics.

[CR53] Veltman F (1996). Defaults in update semantics. Journal of Philosophical Logic.

[CR54] von Fintel K, Keenan E (2019). Determiners, conservativity, witnesses. Journal of Semantics.

[CR55] von Fintelvon Fintel K, Matthewson L (2008). Universals in semantics. The Linguistics Review.

[CR56] Zeijlstra H (2011). On the syntactically complex status of negative indefinites. The Journal of Comparative Germanic Linguistics.

